# How can actuarial science contribute to the field of health technology assessment? An interdisciplinary perspective

**DOI:** 10.1017/S0266462324004781

**Published:** 2025-01-06

**Authors:** Oscar Espinosa, Michael Drummond, Ellyn Russo, David Williams, Donna Wix

**Affiliations:** 1 Economic Models and Quantitative Methods Research Group (IMEMC), Centro de Investigaciones para el Desarrollo, Universidad Nacional de Colombia, Bogotá, D.C., Colombia; 2 Centre for Health Economics, University of York, York, UK; 3 Milliman, Inc., Hartford, CT, USA

**Keywords:** actuarial judgment, risk management, decision models, health technology assessment, modeling, artificial intelligence

## Abstract

A reflective analysis is presented on the potential added value that actuarial science can contribute to the field of health technology assessment. This topic is discussed based on the experience of several experts in health actuarial science and health economics. Different points are addressed, such as the role of actuarial science in health, actuarial judgment, data inputs and their quality, modeling methodologies and the use of decision-analytic models in the age of artificial intelligence, and the development of innovative pricing and payment models.

## Introduction

Recent years have seen an increase in the use of health technology assessment (HTA) by national governments to make decisions regarding investments in their health systems (1). HTA is considered as a methodological bridge between scientific evidence and public health policy with the aim of optimizing the budget allocated to the prevention, treatment, and rehabilitation of diseases (2). According to the current HTA definition, HTA is considered as an interdisciplinary process that aims to determine the value of a health technology (e.g., medicine, health procedure, medical device, etc.), at different points in its life cycle (3).

HTA makes use of explicit methods such as assessments of effectiveness and patient safety studies, health economic evaluation (EE), and budget impact analysis (BIA), with the aim of prioritizing health care investments in society to promote an equitable, high quality, and efficient health system, also including consideration of ethical, legal, social, and implementation aspects (3). It is in the EE and BIA, the economic and financial component of HTA, where we believe that actuarial science – which analyses and assesses the different risks that can materialize in financial impacts, applying mathematical and statistical methods (4–7) – has a key role to play and can bring added value at different methodological points if not already incorporated.

In the following, we will discuss potential contributions that could be made from actuarial science to HTA. Those conducting EE understand, and in many cases have attempted to resolve, several of the issues presented here. However, in reviewing the state of the literature and based on our experience, we see that on the points mentioned here, there are opportunities for which the addition of actuarial expertise can serve to improve practice.

## The role of actuarial science

Actuarial science is the study of the use/application of mathematical and statistical methods to assess and solve financial risks in insurance. Actuarial science applies the mathematics of probability and statistics to define, analyze, and solve the financial implications of uncertain future events. As HTA practitioners are increasingly being called upon to assess new technologies with high levels of uncertainty in their harms, benefits, and costs, we believe actuaries can provide increased insight to complement economic science. Each discipline contributes key information to decision-makers on how to manage financial risk and uncertainty while optimizing patient outcomes (8;9).

In seeking to faithfully represent the complex realities of health technologies, leverage the use of real-world evidence, incorporate elements such as equity and social justice, among other contemporary challenges, the literature has seen a significant acceleration in the development of increasingly complex analytical models to study cost-effectiveness (10;11).

Health actuaries complement analyses with historical experience analysis and benchmarking, implementing predictive models as a risk management tool to establish reimbursement for all aspects of health care (12–14). Importantly, health actuaries combine these evaluations to reach an understanding of the current prevalence and incidence of a condition for which a new technology serves to intervene, the extent to which the new technology will replace other technology(ies) currently in the market, and the options for contracting arrangements that are feasible for the new technology. They also carefully consider the subpopulations, in their analysis as reimbursement levels, incremental cost-effectiveness ratios (ICERs), and other measures will vary substantially.

Pricing has been a long-standing dilemma in the field of health economics. Emerging value-based pricing approaches for health technologies complicate the challenge (15;16). The approaches, ranging from traditional fee-for-service payments to discounts off billed charges, or average wholesale price to prospective payment systems (e.g., bundled arrangements), risk-based payments (e.g., shared savings arrangements), and population-based capitation, seek to increase the potential for cost containment or cost reduction. Thus, in order for reimbursement to a provider or supplier to increase, an entity must absorb more financial risk. Actuaries, who should be involved in any process where there is exposure to risk, can model and help compare the impact of different reimbursement options, which can dramatically affect the results of a health technology appraisal.

Claxton et al. share a possible solution to this problem, which would be to negotiate on the basis of a price where the incremental cost-effectiveness ratio (ICER) is equal to the cost-effectiveness threshold (CET) for the country (15). However, not all countries have a CET estimated with real-world evidence. In this context, actuarial science could help to provide alternative pricing methodologies, for example, from a median ICER or seventy-five percent percentile ICER, using methods that implicitly contain a potential financial risk associated with the CET (8;17). Buckle and Serre have made progress in exploring alternative methodological approaches to new HTAs, which offer valuable insights into the management of financial uncertainty about future reimbursement (18).

This is not an easy process, but the pricing expertise of actuaries can go some way to assist in price formation in health systems. On the other hand, a novel and recent approach, proposed by Paulden (16) from a health economics perspective, but with an important vision of financial economics and risk management, develops a fair pricing framework based on the risk taken by the various parties (e.g., the manufacturer’s risk when developing medicines, payer’s risk when reimbursing medicines, determination of equalizing risk-adjusted rates of return, among other factors of interest).

## Actuarial judgment

While most HTA publications present the basic assumptions of the study, the assumptions of the modeling developed should be made more explicit, according to the context of the health system, current clinical practices, and sector regulation (19). The HTA team should consider the selected perspective^1^, and in doing so, assess the extent to which it is appropriate to adjust the assumptions or methodology to compensate for known deficiencies in the available information systems (20). Researchers must therefore know when and how certain assumptions – implicit or explicit – or methodologies should be used, knowing that these have the potential to underestimate or overestimate results (20).

Furthermore, it must be assessed whether all assumptions are reasonable and appropriate as a whole, as they may be logical at the individual level, but unreasonable in the aggregate, rendering the proposed approach invalid. The review of the underlying parameters of the decision analytical model, together with the internal consistency of the assumptions and methodology, will allow significant interdependencies to be modeled appropriately (4;19–21). This is a particular strength of actuarial science, for which known differences in existing information, or information that is otherwise lacking, is not an infrequent occurrence (20).

Actuaries are required to adhere to strict professional and ethical standards to ensure users can rely on the product of their work. This includes determining if underlying data are sufficient and reliable for their purpose, to validate that the data are consistent, complete, and accurate, and to disclose modifications made to the data to serve their purpose. Other standards of practice relating to HTA include Risk Classification (ASOP12), Use of Health Status Based on Risk Adjustment Method (ASOP45), Credibility Procedures (ASOP25), Social Insurance (ASOP32), and Modeling (ASOP56) (20). Some actuaries even implement peer review standards for the work they produce, subjecting their work to the scrutiny of one or more colleagues of similar or higher credentials.

Regarding the main sources of consultation and extraction of epidemiological data for effectiveness and safety, clinical judgment is undoubtedly fundamental, as it is particularly necessary for determining clinically meaningful outcomes differences. Actuarial judgment can augment this further by providing empirically established statistical links between patient data and outcomes, particularly as they relate to cost information, and attempts to avoid cognitive biases in expert opinion (22;23). Several studies have demonstrated the possible synergy of taking into account the two criteria (clinical and actuarial), for example, in the field of psychiatry (22;24;25). However, there may also be certain biases in this approach, that are well described by Tredger et al. (26). A first step is the recent paper by Bojke et al. (27), funded by the NIHR HTA program, which addresses this issue by establishing a structured protocol for expert consultation to inform health care decision making, drawing, among other disciplines, on the important contributions that have been made from actuarial work.

Finally, actuarial science, because of its deep involvement with the business sector, has basic principles of effective and assertive communication to convey complex information to decision-makers, especially from a corporate perspective (28). In a results section and considering the perspective chosen by the HTA researchers, actuaries can help make clear what the objectives of the study are, explain what is modeled and what is not, and how the decision analytic model has been validated. Moreover, actuaries will clearly articulate the researcher’s assumptions for the value from various stakeholders’ perspective, describing the assumption and any sensitivity analysis performed, making explicit the limitations and uncertainties of any analytical model used for the assessment and its implications (14). It is for all of these reasons that actuaries can make important additions to HTA research teams.

## Data inputs and their quality

There are different applications of costing in health economics, including estimating the costs of universal health coverage at the country level (29), projecting health costs in the short term (30), or measuring the impact of the COVID-19 pandemic on future health care costs (31), based on an observed age structure for health insurance claim frequencies, annual aggregate losses, and mortality dynamics. Typically, costs are estimated from the perspective of the decision-maker concerned. Actuaries may provide insights into relevant perspectives within the context of the health system(s) in which an HTA is being performed; that is, in identifying costs according to who will benefit (32), and how this differs depending on the component of the health care system responsible for the costs (33). An HTA performed in the context of a universal health system, for example, is currently limited by the lack of literature on the costs of universal health systems. Similarly, HTAs performed using specific populations (from a private perspective) must adequately represent the insurance segment for which the evaluation is intended, or sufficiently capture the various insurance segments, as costs vary widely by insurance across health care services.

When using real-world data to evaluate the effectiveness of a health technology, data quality matters. Data audits can also be employed for analyses as needed, though these are not often done or required for real-world evidence in HTA. Nevertheless, the potential for biases due to data inconsistency, incompleteness, and/or inaccuracy can subsequently lead to misinformed results and subsequent decision-making. Actuarial Standard of Practice Number 23 provides guidance to actuaries for selecting data, including reliance on data supplied by others (34). Actuaries are instructed to:

*(…) make a reasonable effort to identify data values that are questionable or relationships that are significantly inconsistent. If the actuary believes questionable or inconsistent data values could have a significant effect on the analysis, the actuary should consider taking further steps, when practical, to improve the quality of the data. The actuary should disclose in summary form any unresolved questionable data values that the actuary believes could have a significant effect on the analysis, in accordance with section 4.1(d). The actuary also should disclose any significant steps the actuary has taken to improve the data, in accordance with section 4.1(e).* (34)

This vision could strengthen the already meritorious advances that the *Real World Evidence & Artificial Intelligence* interest group within HTA International (HTAi) has been working on since 2020 (35). The importance of communicating and disclosing findings from data reviews, including enhancements due to a potential or probable issue(s) in the data, is critical to the actuary’s responsibility as is identifying something in the first place.

## Modeling methodologies and the use of decision-analytic models in the AI era

Modeling in the context of EE requires methodological choices and a number of assumptions. Actuaries may offer a different perspective on how these choices or assumptions are made. For example, a frequent problem observed in the different health information systems is under-diagnosis. As diseases are under-diagnosed at different levels of the health system (i.e., they are not diagnosed or are diagnosed with a significant time lag), in addition to the multiple disadvantages for patients, future health economics modeling is affected, given that it is usually based on these data that the proposed modeling for reimbursement decisions, pricing, and so forth is carried out. Recently, in December 2023 Stocking et al. (36) proposed a robust and novel actuarial methodology that uses longitudinal records of diagnoses based on administrative (claims) data to identify latent or undiagnosed members who are highly likely to have a chronic condition. This type of conceptual framework could contribute to the more accurate estimation of chronic disease prevalence and thus to the analysis of real-world evidence so prevalent in recent HTA analyses.

Conversely, with the boom in artificial intelligence (AI), clinical information is becoming increasingly important for improving the quality of analytics. While data scientists are making impressive advances in real-time data collection and monitoring, this requires an interpretive and usable framework for HTAs. We therefore agree with Duncan et al. (37), who argue that outcomes are likely to be suboptimal if complex AI tools applied to health rely solely on the work of data scientists. The particular skills and specific expertise of health economists and health actuaries, such as risk analysis and behavioral economics, are needed to maximize the value of the use, design, and implementation of economic analyses and budget impact analyses. At this point, while there may be an overlap between the skills of actuaries and economists, these two professions bring complementary and essential skill sets to an analytical team in the age of AI.

## Development of innovative pricing and payment models

A substantial proportion of the new therapies being subjected to HTA are advanced therapy medicinal products, including cell and gene therapies. A key challenge to HTA is that these therapies represent a large up-front cost, but have benefits that stretch over a period of many years and are uncertain. One proposed solution to these challenges is to develop a pricing and payment model that spreads the payments over a number of years, in the form of an annuity or subscription (38).

This is an area where actuaries can offer a different and distinct perspective, by contributing to the design of the different payment models and demonstrating the level of benefits and risks that different approaches attribute to the various parties in the pricing and payment agreement.

## Conclusion

Although the field of HTA has evolved rapidly in recent decades, there are opportunities for improvement that could enhance the development of these technical studies. As an interdisciplinary process, the HTA field can learn from common practices in actuarial analysis to strengthen the application and usefulness of analytical decision models. For example, some countries, such as Colombia, have begun to incorporate actuarial profiles into HTA development groups, strengthening the scientific analysis for the health system (39). This resulted, for example, in the successful design, development, and implementation of disruptive analyses such as the optimization of the COVID-19 vaccine portfolio, among other projects (40).

Almost a decade ago, Bath wrote an important text that is still valid today (41), health economics will have to continue to evolve and rely as best it can on other disciplines, given that its challenges will be increasingly complex to address in hyper-connected health systems under great pressure both from the demand for services and from industrial supply and innovation. Experience, judgment, heuristics, and robust quantitative modeling are essential for sound decision-making under conditions of uncertainty.

## Footnotes

1 Sometimes (mostly outside academia) the perspective of the economic models used in HTA reports is not chosen by those doing the reporting, but is defined a priori, often by national guidelines.
